# Remarkable sequence similarity between the dinoflagellate-infecting marine girus and the terrestrial pathogen African swine fever virus

**DOI:** 10.1186/1743-422X-6-178

**Published:** 2009-10-27

**Authors:** Hiroyuki Ogata, Kensuke Toyoda, Yuji Tomaru, Natsuko Nakayama, Yoko Shirai, Jean-Michel Claverie, Keizo Nagasaki

**Affiliations:** 1 Information Génomique et Structurale, CNRS-UPR2589, Institut de Microbiologie de la Méditerranée, Parc Scientifique de Luminy, Aix-Marseille Université, 163 Avenue de Luminy, Case 934, 13288 Marseille Cedex 9, France; 2 Harmful Algal Bloom Division, National Research Institute of Inland Sea, Fisheries Research Agency, 2-17-5 Maruishi, Hatsukaichi, Hiroshima 739-0452, Japan

## Abstract

*Heterocapsa circularisquama *DNA virus (HcDNAV; previously designated as HcV) is a giant virus (girus) with a ~356-kbp double-stranded DNA (dsDNA) genome. HcDNAV lytically infects the bivalve-killing marine dinoflagellate *H. circularisquama*, and currently represents the sole DNA virus isolated from dinoflagellates, one of the most abundant protists in marine ecosystems. Its morphological features, genome type, and host range previously suggested that HcDNAV might be a member of the family *Phycodnaviridae *of Nucleo-Cytoplasmic Large DNA Viruses (NCLDVs), though no supporting sequence data was available. NCLDVs currently include two families found in aquatic environments (*Phycodnaviridae*, *Mimiviridae*), one mostly infecting terrestrial animals (*Poxviridae*), another isolated from fish, amphibians and insects (*Iridoviridae*), and the last one (*Asfarviridae*) exclusively represented by the animal pathogen African swine fever virus (ASFV), the agent of a fatal hemorrhagic disease in domestic swine. In this study, we determined the complete sequence of the type B DNA polymerase (PolB) gene of HcDNAV. The viral PolB was transcribed at least from 6 h post inoculation (hpi), suggesting its crucial function for viral replication. Most unexpectedly, the HcDNAV PolB sequence was found to be closely related to the PolB sequence of ASFV. In addition, the amino acid sequence of HcDNAV PolB showed a rare amino acid substitution within a motif containing highly conserved motif: YSDTDS was found in HcDNAV PolB instead of YGDTDS in most dsDNA viruses. Together with the previous observation of ASFV-like sequences in the Sorcerer II Global Ocean Sampling metagenomic datasets, our results further reinforce the ideas that the terrestrial ASFV has its evolutionary origin in marine environments.

## Findings

Dinoflagellates (Dinophyceae) are one of the highly abundant and ubiquitous unicellular eukaryotic ("protistan") components in marine environments [1]. They constitute a major class of eukaryotes within the Alveolata, a firmly established deep phylogenetic lineage that includes other diverse classes of protists, such as apicomplexans and ciliates [2]. Some dinoflagellates are autotrophic using photosynthesis, some are heterotrophic using endocytotic feeding, and many dinoflagellates are mixotrophic having both modes of nutrition. Blooms of certain photosynthetic dinoflagellates kill fish and bivalves, or pollute shellfishes for food with particular toxins, and can lead to serious economic damages in aquaculture [3,4]. *Heterocapsa circularisquama *forms blooms causing massive death of shellfish such as pearl oysters and mussels, and is one of the most intensively studied dinoflagellate species [5].

HcDNAV is a marine giant virus (or "girus" [6,7]) containing dsDNA genome, and lytically infects *H. circularisquama *[8,9]. HcDNAV is considered to play a significant role in the demise of *H. circularisquama *blooms [9,10]. HcDNAV has a large icosahedral capsid (180-210 nm in diameter), which packs a ~356-kbp genome [8,11]. During its multiplication, virions emerge from a specific cytoplasm compartment, called "viroplasm", which is created by the virus [9]. HcDNAV is the sole DNA virus currently isolated from dinoflagellates, and to our knowledge, is the only DNA virus isolated from the superphylum Alveolata [12]. Based on its host range, genome type/size and microscopic features, HcDNAV was previously suggested to be a member of *Phycodnaviridae *[13]. However, there has been no molecular data supporting this tentative classification.

*Phycodnaviridae *includes intensively-studied algal virus members such as chlorella viruses and *Emiliania huxleyi *viruses [14-17], and belongs to a larger group of eukaryotic DNA viruses called NCLDVs [18]. NCLDVs complete their replication cycle within the host cytoplasm, and share an array of conserved core genes for transcription, RNA processing, replication, DNA packaging, and structural components. Other viral families of NCLDVs are *Mimiviridae*, *Poxviridae*, *Iridoviridae*, and *Asfarviridae*. *Mimiviridae *is represented by the freshwater amoeba-infecting mimivirus [19] and its close relative mamavirus [20]. Based on the sequences of PolB, the most conserved NCLDV core genes, three algal viruses have been suggested to belong to *Mimiviridae *[21]. *Poxviridae *include a number of successful pathogens known to infect a tremendous variety of terrestrial animals, such as insects, reptiles, birds, and mammals [22]. Iridoviruses infect invertebrate and cold-blooded vertebrate hosts, and includes numerous emerging pathogens of fishes and amphibians [23]. The last family *Asfarviridae *[24,25] is currently represented by a sole species, African swine fever virus (ASFV) with a 170 kbp dsDNA genome [26]. ASFV is a large (~200 nm in diameter), intracytoplasmically-replicating arbovirus, naturally maintained in a sylvatic cycle between wild swine (warthogs and bushpigs) and argasid ticks (*Ornithodoros*). In these hosts, ASFV infection is usually asymptomatic [27]. However, ASFV causes an acute hemorrhagic infection in domestic swine with mortality rates up to 100% for some viral isolates.

In an attempt to further characterize HcDNAV, we performed a low coverage shotgun sequencing of its genome. Specifically, from 4 liters of HcDNAV suspension (lysate of HcDNAV-infected *H. circularisquama *on 6 dpi), virus particles were collected as described in [11]. The viral genomic DNA was purified in a PFGE-gel and was subjected to shotgun sequencing (coverage = 0.11 X). Resulting sequence reads covered part of the region containing a PolB-like sequence. With the use of tail-PCR method [28], we successfully determined a 5,800 bp sequence (DDBJ accession number AB522601) containing an open reading frame (ORF) for the complete HcDNAV PolB gene. By means of a reverse transcription-PCR (RT-PCR) experiment, the PolB gene was shown to be transcribed to mRNA (additional file 1); thus, it is most likely crucial for the replication of HcDNAV.

HcDNAV PolB gene was found to be 3,675 bp long (forward strand, position = nt 1,913-5,590 in AB522601), punctuated by normal start and stop codons, and no intron or intein-like sequence was observed. The predicted protein product is 1,225 amino acids (aa) long. Unexpectedly, the translated amino acid sequence showed the closest BLASTP hits against PolB sequences from different ASFV isolates, with the best homolog being DPOL_ASFL6 (identity = 27%, bit score = 311, E-value = 4.10E-82) in the NCBI non-redundant sequence database. The best non-ASFV hit corresponded to the PolB sequence of *Pyramimonas orientalis *virus (DPOL_POV01, identity = 23%, bit score = 131, E-value = 4.10E-28). A multiple sequence alignment of the HcDNAV PolB and its close homologs confirmed the presence of conserved residues for exonuclease and polymerase activities [29] (additional file 2). Curiously, the HcDNAV PolB sequence exhibited a rarely observed amino acid substitution within the motif containing two highly conserved metal binding aspartic acid residues; HcDNAV exhibits the motif YSDTDS- instead of the YGDTDS- sequence usually found in dsDNA viruses. In addition, we identified two ORFs in the upstream region of the PolB ORF in a divergent orientation. Their products were respectively predicted to be 245 and 194 aa in length (positions = nt 463-1,200 and 1,255-1,839). The former showed a significant similarity to HNH endonucleases with its BLASTP best hit to mimivirus L245 (YP_142599, E-value = 4E-11); the latter showed a significant similarity to hypothetical proteins from NCLDVs with its best hit to mimivirus R325 (annotated as a metal-dependent hydrolase, YP_142679.1, E-value = 1E-12). Incidentally, R325 is located near the PolB gene (R322) in the mimivirus genome [30].

**Figure 1:**
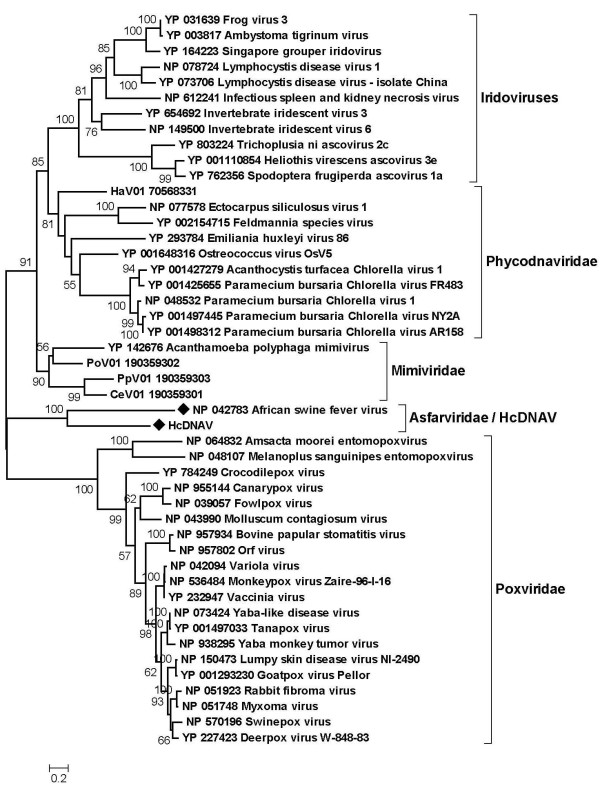
**Maximum likelihood tree of PolB amino acid sequences from NCLDVs**. Alignment was constructed with the use of T-Coffee. All the gap-containing amino acid residue sites were removed before tree construction. The phylogenetic tree was constructed using PhyML [38] available at Phylogeny.fr [39] using WAG matrix and gamma distribution. Branch labels indicate bootstrap percentages (≥ 50%) after 100 replicates. The tree is essentially an unrooted tree, albeit mid-point rooted only for presentation purpose. The same method was used for the phylogenetic trees in Fig. 2, Fig. 3 and in the additional file 3. HcDNAV and ASFV sequences are indicated by filled diamond marks. CeV: *Chrysochromulina ericina *virus; PoV: *Pyramimonas orientalis *virus; HaV: *Heterosigma akashiwo *virus.

**Figure 2:**
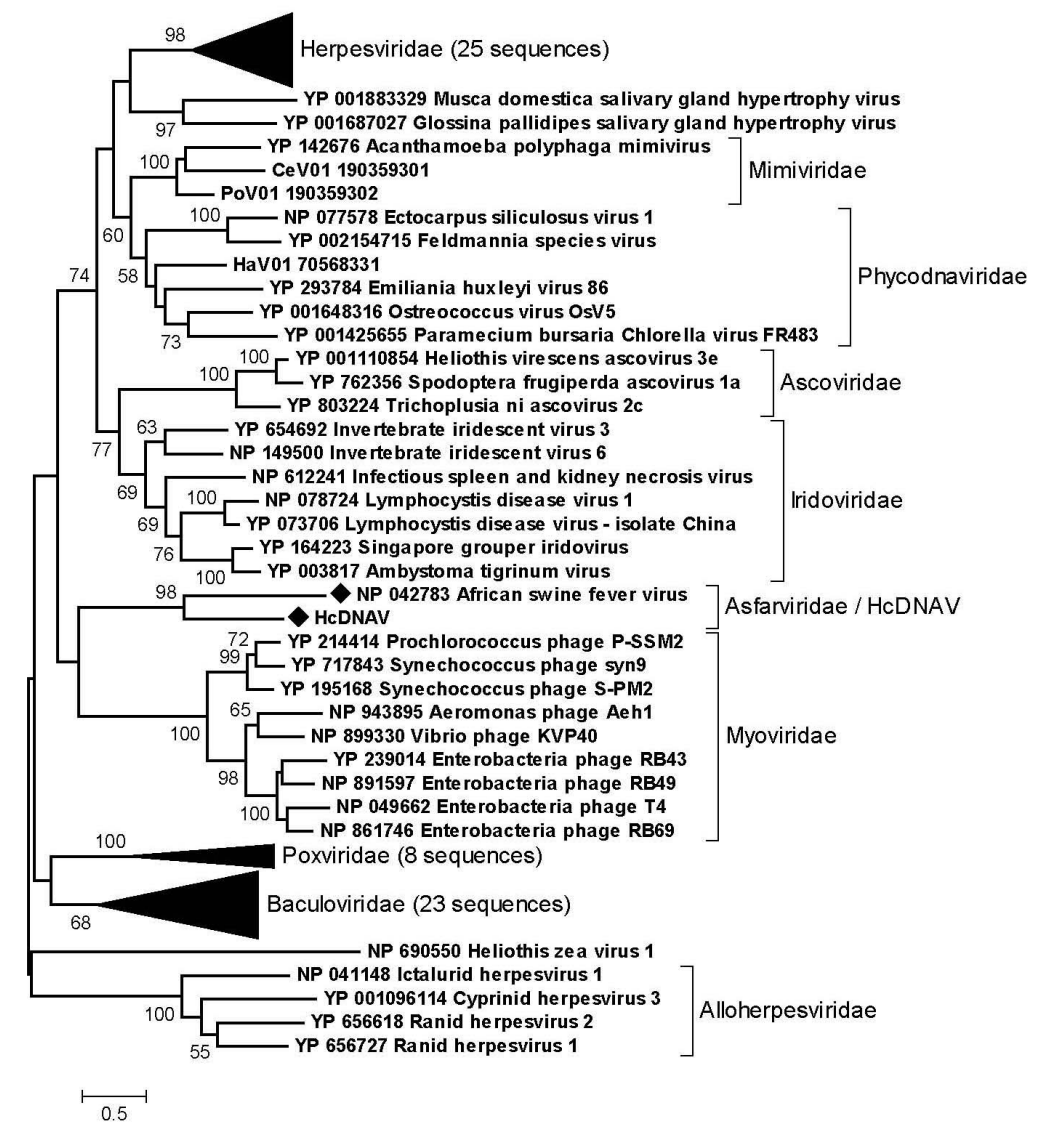
**Maximum likelihood tree of PolB amino acid sequences from diverse groups of viruses**. HcDNAV and ASFV sequences are indicated by filled diamond marks.

To examine the unexpected sequence similarity between the HcDNAV and ASFV PolBs, we conducted a series of maximum likelihood phylogenetic analyses. First, we aligned the HcDNAV PolB sequence with its homologs from NCLDVs. A phylogenetic tree based on the 362 amino acid residue sites from the alignment supported the monophyletic grouping of HcDNAV and ASFV with a 100% bootstrap value (Fig. 1). The grouping of each of the other four NCLDV families was also supported by a high bootstrap value (100% for *Iridoviridae*, 81% for *Phycodnaviridae*, 90% for *Mimiviridae *and 100% for *Poxviridae*). Next, we used a wider range of viral homologs including those of bacteriophages. The resulting tree based on 320 amino acid residues again supported the grouping of HcDNAV/ASFV with a 98% bootstrap value (Fig. 2).

In addition, we obtained a short sequence partially corresponding to an RNA polymerase II large subunit gene from HcDNAV genomic DNA (AB522602), for which we obtained a similar result. The 892 bp sequence showed BLASTX best hit against ASFV RNA polymerase sequence (RPB1_ASFM2, E-value = 2E-12). A monophyletic grouping between the HcDNAV sequence (97 aa) and the ASFV RNA polymerase sequence was again received a high bootstrap value of 87% (additional file 3).

Our homology search and phylogenetic analyses thus confirm that the newly determined HcDNAV sequences are most closely related to their ASFV homologs. This result is in clear contradiction with the previous proposal that HcDNAV may belong to the *Phycodnaviridae *[13].

**Figure 3:**
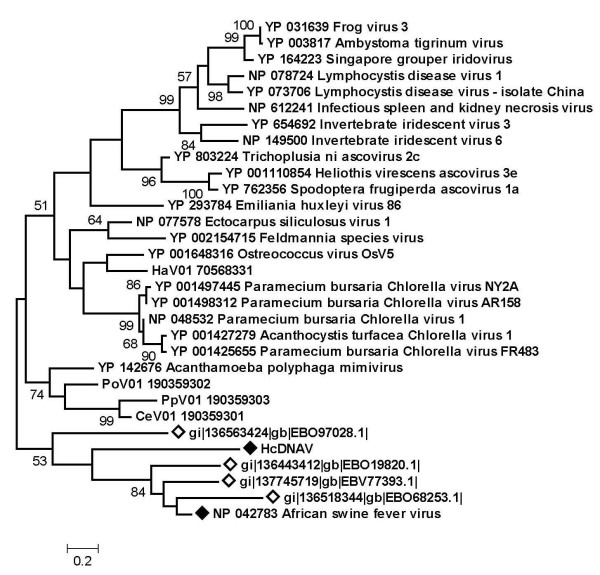
**Maximum likelihood tree of PolB amino acid sequences from NCLDVs and several sequences from environmental samples (indicated by open diamond marks)**. HcDNAV and ASFV sequences are indicated by filled diamond marks.

A previous "phylogenetic mapping" survey of the metagenomic sequence data sets generated by the Global Ocean Sampling (GOS) expedition [31] revealed several PolB-like sequences most closely related to the PolB sequence of ASFV [32]. This observation suggested the presence of ASFV-related viruses in marine environments. In order to examine whether the "ASFV-like" marine PolB sequences were close to the HcDNAV PolB sequence, we retrieved 267 sequences from the environmental sequence collection of NCBI/GenBank using the PolB sequences of HcDNAV and ASFV as queries (E-value < 1E-10). These environmental sequences were in turn searched against the NCBI non-redundant sequence database and the HcDNAV PolB sequence. Of the 267 sequences, 15 showed their best hit to the ASFV PolB, one showed its best hit to HcDNAV (gi|136563424), and the remaining sequences had their best hit to other viruses or cellular organisms. Therefore, most of the ASFV/HcDNAV-like PolB sequences in the marine environmental collection are more closely related to the ASFV PolB than to the HcDNAV homolog. A phylogenetic tree using several environmental sequences supported their grouping with the terrestrial ASFV PolB (bootstrap value = 84%, Fig. 3).

PolB is one of the most reliable phylogenetic markers for large eukaryotic DNA viruses [32,33]. The fact that the HcDNAV PolB was not grouped with the PolBs from phycodnaviruses strongly argues against the previous tentative classification of HcDNAV in the *Phycodnaviridae *family [13]. It is clear that the definitive classification of HcDNAV will require the complete sequencing of its genome. It may also turn out that the HcDNAV genome corresponds to a mosaic of NCLDV genes with different evolutionary histories, precluding a simple classification scheme. Pending its complete genome sequencing, we recently proposed to the ICTV to create a new genus "Dinodnavirus" where to tentatively classify the HcDNAV.

Our finding now establishes an evolutionary link between a terrestrial pathogen and a marine girus. A recent metagenomic analysis of corals provided evidence for the existence of viruses related to herpesviruses [34], which have been mostly isolated as pathogens of terrestrial animals. So far, giruses of 7 algal classes [12,35] have been isolated; still, we know next to nothing about viruses infecting other protists in aquatic environments. Given the huge diversity of protists [36,37], a comparable diversity probably exists for marine viruses living in these environments. Exploring this hidden viral world is necessary to our understanding of the evolutionary relationships between aquatic viruses and their terrestrial relatives.

## Competing interests

The authors declare that they have no competing interests.

## Authors' contributions

NK conceived the study. YS, KT and NN conducted purification and sequencing of HcDNAV and RT-PCR experiment. HO designed and carried out bioinformatics analyses. HO, JMC, YT and NK contributed to the interpretation of data and wrote the manuscript. All authors read and approved the final manuscript.

## Supplementary Material

Additional file 1 **Transcription of the PolB gene of HcDNAV**. To verify the transcription of the HcDNAV PolB gene, reverse transcription-PCR (RT-PCR) experiment was conducted. The total RNA samples were isolated from HcDNAV-inoculated *Heterocapsa circularisquama *cells collected at 0, 1, 6, 12, and 24 hpi, then reverse-transcribed according to the method given by Nagasaki et al. [10]. PCR amplification was performed using a DNA polymerase KOD FX (Toyobo, Osaka, Japan) and primers designed for amplification of HcDNAV PolB gene fragment (01F: ACG TTT TAA ATG ATG TTA TTA ATG, 01R: GCC ATT TTA ATA TAT GAA TAA A); the reaction cycling conditions were 94°C for 2 min, then 25 cycles of 98°C for 10 s, 50°C for 30 s, 68°C for 1 min. Lanes 1 to 5 show the RT-PCR fragments amplified from cDNAs at 0, 1, 6, 12, and 24 hpi, respectively, and lane 6 shows the PCR fragments amplified from HcDNAV DNA (positive control). Click here for file

Additional file 2 **Conserve blocks from the multiple sequence alignment of PolB sequences from NCLDVs**. The data provided shows the presence of conserved residues for exonuclease and polymerase activities in the HcDNAV PolB and its close homologs. Species abbreviation is followed by a database sequence identifier. Intein sequences were removed from the sequences prior to alignment. The alignment was generated by T-Coffee [40] and ClustalX [41]. AmEPV: *Amsacta moorei *entomopoxvirus 'L'; APMV: Acanthamoeba polyphaga mimivirus; ASFV: African swine fever virus; CeV: *Chrysochromulina ericina *virus; EhV: *Emiliania huxleyi *virus 86; EsV: *Ectocarpus siliculosus *virus 1; FsV: *Feldmannia *species virus; HaV: *Heterosigma akashiwo *virus 01; HcDNAV: *Heterocapsa circularisquama *DNA virus; HvAv: *Heliothis virescens *ascovirus 3e; IIV: Invertebrate iridescent virus 6; LCDV: Lymphocystis disease virus 1; OtV: *Ostreococcus *virus OsV5; PBCV: *Paramecium bursaria Chlorella *virus 1; PoV: *Pyramimonas orientalis *virus; VAR: Variola virus. Click here for file

Additional file 3 **Maximum likelihood tree of RNA polymerase large subunit amino acid sequences**. The tree is based on an alignment containing 66 amino acid residues with no gaps. Alignment and tree construction method is the same as those used for the tree in Fig. 1. HcDNAV and ASFV sequences (indicated by filled diamond marks) are grouped and supported by a bootstrap of 87%. Click here for file
