# Supplementary figures and images for: Remarkable sequence similarity between the dinoflagellate-infecting marine girus and the terrestrial pathogen African swine fever virus
Source: Virol J. 2009 Oct 27;6:178. doi: 10.1186/1743-422X-6-178 (PMC2777158; doi:10.1186/1743-422X-6-178)

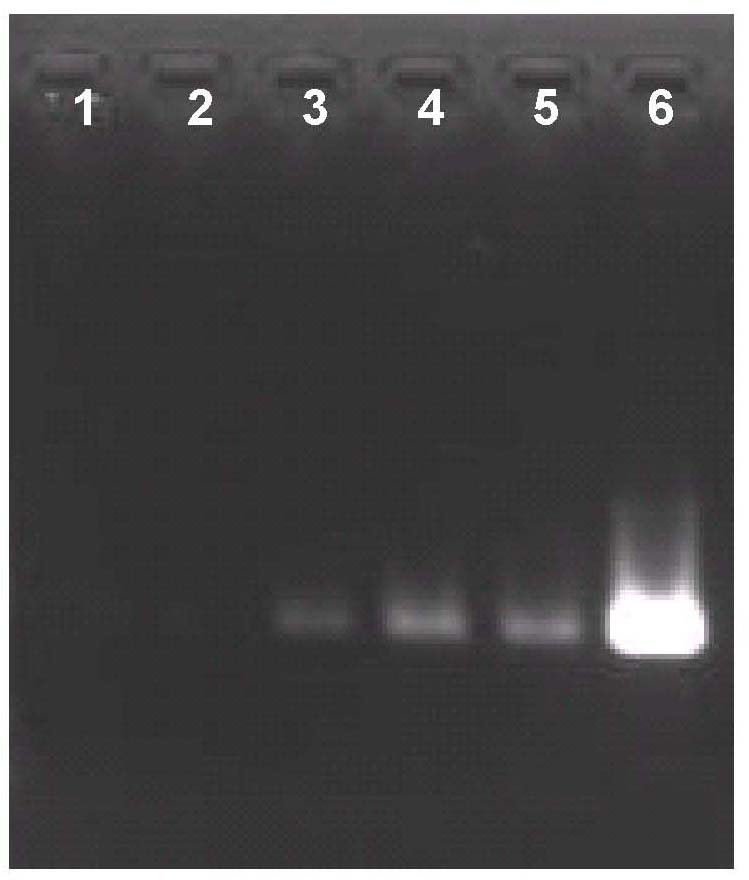

Supplement: Additional file 1 — Transcription of the PolB gene of HcDNAV. To verify the transcription of the HcDNAV PolB gene, reverse transcription-PCR (RT-PCR) experiment was conducted. The total RNA samples were isolated from HcDNAV-inoculated Heterocapsa circularisquama cells collected at 0, 1, 6, 12, and 24 hpi, then reverse-transcribed according to the method given by Nagasaki et al. [10]. PCR amplification was performed using a DNA polymerase KOD FX (Toyobo, Osaka, Japan) and primers designed for amplification of HcDNAV PolB gene fragment (01F: ACG TTT TAA ATG ATG TTA TTA ATG, 01R: GCC ATT TTA ATA TAT GAA TAA A); the reaction cycling conditions were 94°C for 2 min, then 25 cycles of 98°C for 10 s, 50°C for 30 s, 68°C for 1 min. Lanes 1 to 5 show the RT-PCR fragments amplified from cDNAs at 0, 1, 6, 12, and 24 hpi, respectively, and lane 6 shows the PCR fragments amplified from HcDNAV DNA (positive control). [file 1743-422X-6-178-S1.JPEG]

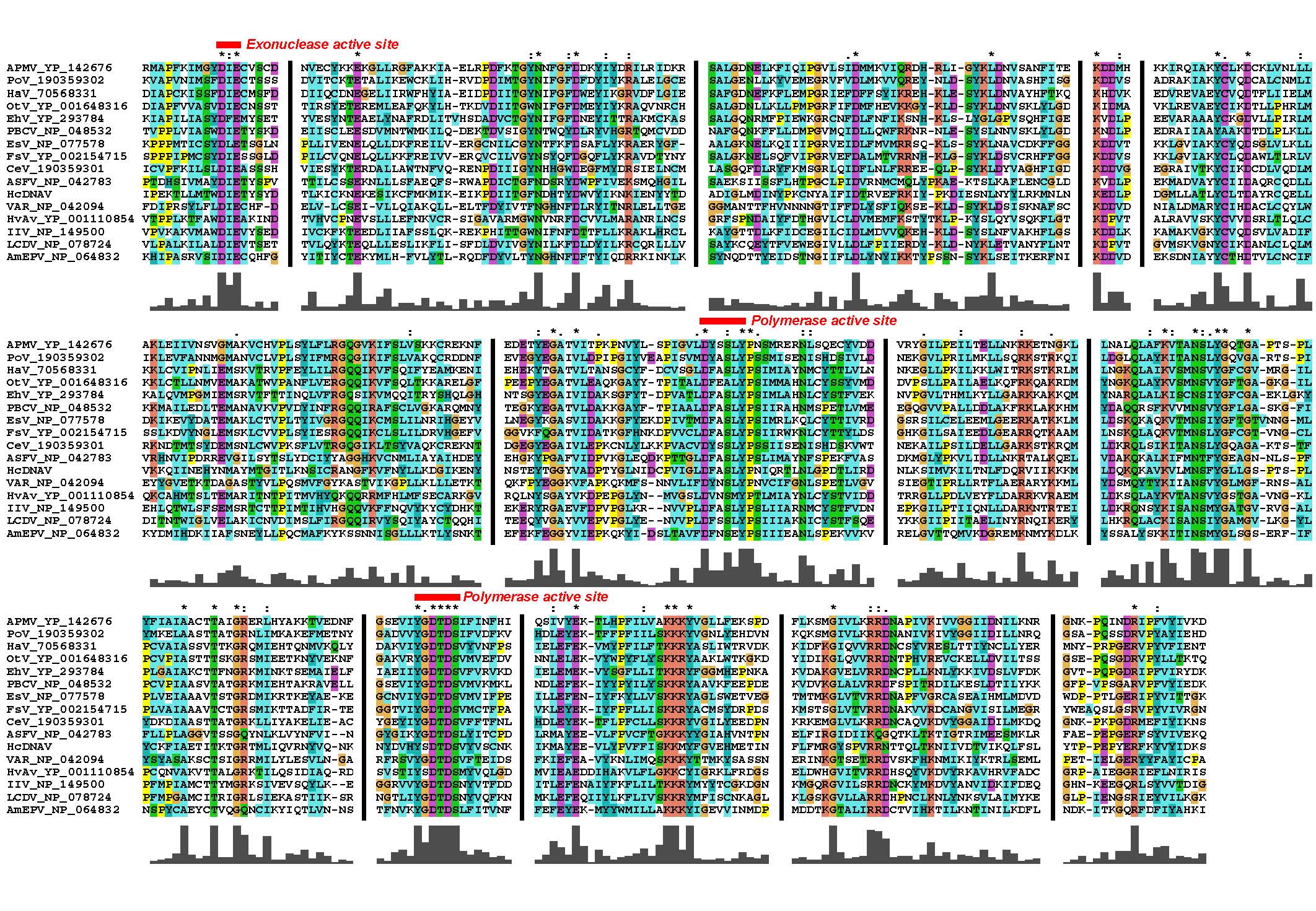

Supplement: Additional file 2 — Conserve blocks from the multiple sequence alignment of PolB sequences from NCLDVs. The data provided shows the presence of conserved residues for exonuclease and polymerase activities in the HcDNAV PolB and its close homologs. Species abbreviation is followed by a database sequence identifier. Intein sequences were removed from the sequences prior to alignment. The alignment was generated by T-Coffee [40] and ClustalX [41]. AmEPV: Amsacta moorei entomopoxvirus 'L'; APMV: Acanthamoeba polyphaga mimivirus; ASFV: African swine fever virus; CeV: Chrysochromulina ericina virus; EhV: Emiliania huxleyi virus 86; EsV: Ectocarpus siliculosus virus 1; FsV: Feldmannia species virus; HaV: Heterosigma akashiwo virus 01; HcDNAV: Heterocapsa circularisquama DNA virus; HvAv: Heliothis virescens ascovirus 3e; IIV: Invertebrate iridescent virus 6; LCDV: Lymphocystis disease virus 1; OtV: Ostreococcus virus OsV5; PBCV: Paramecium bursaria Chlorella virus 1; PoV: Pyramimonas orientalis virus; VAR: Variola virus. [file 1743-422X-6-178-S2.JPEG]

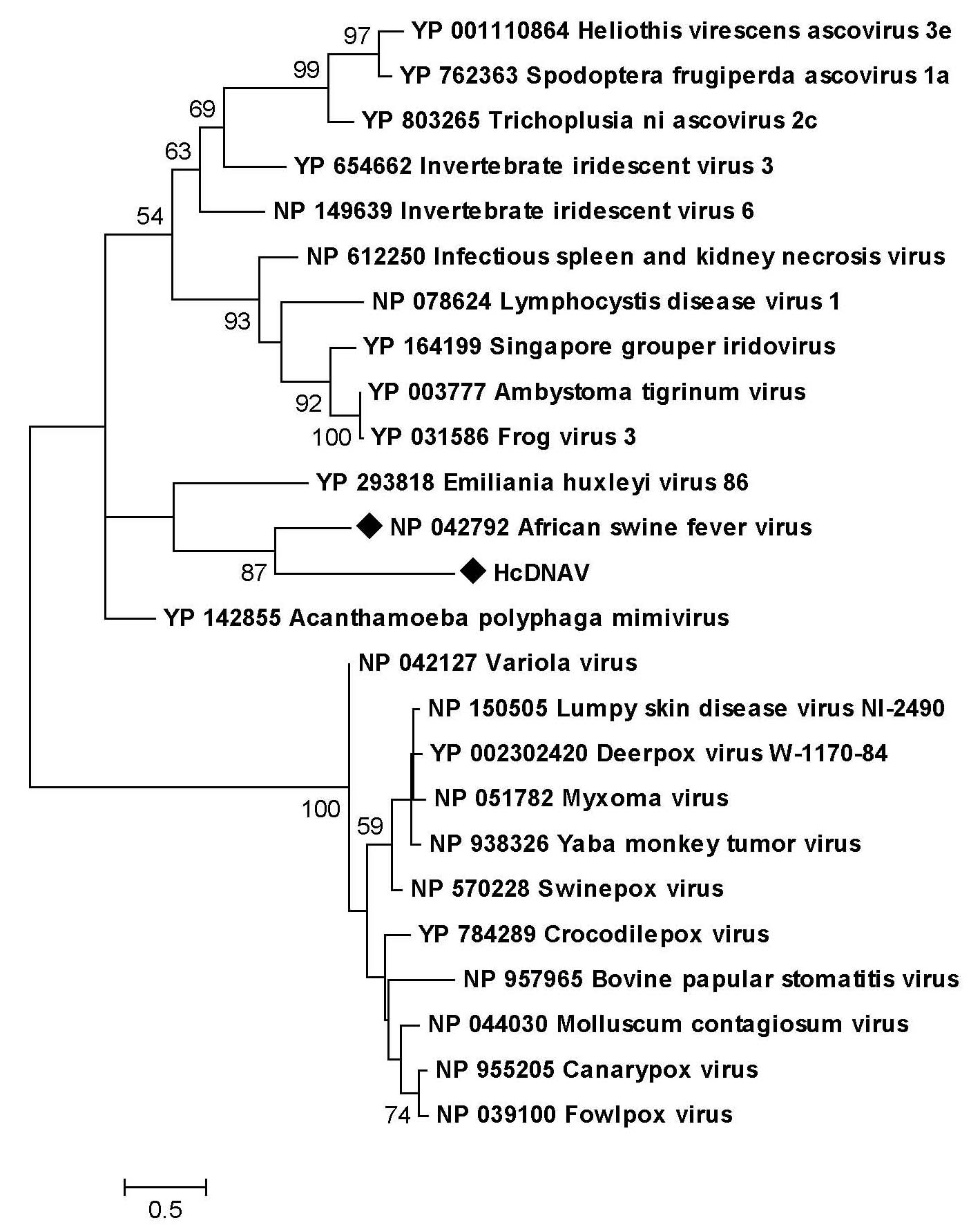

Supplement: Additional file 3 — Maximum likelihood tree of RNA polymerase large subunit amino acid sequences. The tree is based on an alignment containing 66 amino acid residues with no gaps. Alignment and tree construction method is the same as those used for the tree in Fig. 1. HcDNAV and ASFV sequences (indicated by filled diamond marks) are grouped and supported by a bootstrap of 87%. [file 1743-422X-6-178-S3.JPEG]
